# Plasminogen Receptors and Fibrinolysis

**DOI:** 10.3390/ijms22041712

**Published:** 2021-02-08

**Authors:** Lindsey A. Miles, Lina Ny, Malgorzata Wilczynska, Yue Shen, Tor Ny, Robert J. Parmer

**Affiliations:** 1 Department of Molecular Medicine, The Scripps Research Institute, La Jolla, CA 92037, USA; 2 Department of Medical Biochemistry and Biophysics, Umeå University, 90187 Umeå, Sweden; lak15lny@student.lu.se (L.N.); malgorzata.wilczynska0006@gmail.com (M.W.); wyueshen@gmail.com (Y.S.); tor.ny@umu.se (T.N.); 3 Department of Medicine, University of California San Diego, La Jolla, CA 92093, USA and Veterans Administration San Diego Healthcare System, San Diego, CA 92161, USA; rparmer@ucsd.edu

**Keywords:** plasminogen, fibrinolysis, plasminogen receptors

## Abstract

The ability of cells to promote plasminogen activation on their surfaces is now well recognized, and several distinct cell surface proteins have been demonstrated to function as plasminogen receptors. Here, we review studies demonstrating that plasminogen bound to cells, in addition to plasminogen directly bound to fibrin, plays a major role in regulating fibrin surveillance. We focus on the ability of specific plasminogen receptors on eukaryotic cells to promote fibrinolysis in the in vivo setting by reviewing data obtained predominantly in murine models. Roles for distinct plasminogen receptors in fibrin surveillance in intravascular fibrinolysis, immune cell recruitment in the inflammatory response, wound healing, and lactational development are discussed.

## 1. Introduction

The ability of cells to promote plasminogen activation on their surfaces is now well recognized, and several distinct cell surface proteins have been demonstrated to function as plasminogen receptors. Here we review studies demonstrating that plasminogen bound to cells, in addition to plasminogen directly bound to fibrin, has a major role in regulating fibrinolysis. We focus on the ability of specific plasminogen receptors on eukaryotic cells to promote fibrinolysis in the in vivo setting by reviewing data obtained predominantly in murine models.

## 2. Similarities in Plasminogen Activation on Fibrin and on Cell Surfaces

**Figure 1:**
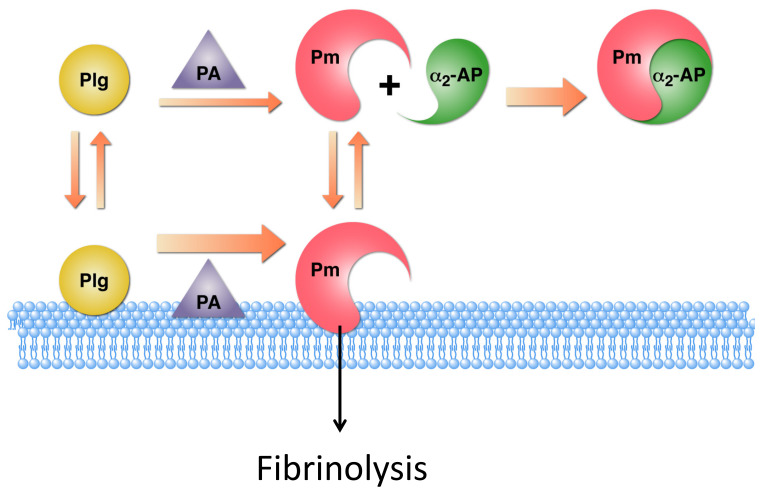
Enhancement of plasminogen activation on the cell surface. Activation of cell-associated plasminogen (Plg) to plasmin (Pm) by cell-associated plasminogen activators (PA) is markedly enhanced compared to the reaction in solution. The Pm formed remains on the cell surface, where it is relatively protected from its inhibitor, α_2_-antiplasmin (α_2_-AP). This results in the association of the proteolytic activity of plasmin with the cell surface to function in fibrinolysis. This figure is modified from a figure originally published in © 2012 Miles, L.A.; Andronicos, N.M.; Chen, E.I.; Baik, N.; Bai, H.; Parmer, C.M.; Lighvani, S.; Nangia, S.; Kiosses, W.B.; Kamps, M.P.; Yates, J.R., III; Parmer, R.J. Published in under CC BY 3.0 license. Available online: http://dx.doi.org/10.5772/31113 [7].

Over 25 years ago, the ability of plasminogen to interact with platelets was first demonstrated [1]. Subsequently, the presence of plasminogen binding sites on virtually every cell type examined (with the exception of red cells) has been established (reviewed in [2]). The interaction of plasminogen with cell surfaces mimics the interaction of plasminogen with fibrin. That is, when plasminogen is bound to cells, its activation is markedly enhanced compared with the reaction in solution reviewed in [3]) and Figure 1. Similarly to plasminogen activation on fibrin [4], this enhancement takes place upon the concomitant localization of plasminogen activators, tissue type plasminogen activator (t-PA) and/or urokinase (u-PA) on the cell surface.

Furthermore, as with the interaction of plasminogen with fibrin, its interaction with eukaryotic cell surfaces is mediated by the lysine binding sites within plasminogen kringles [5] that interact with the carboxyl-terminal lysyl residues exposed on cell surfaces. In additional concordance with plasminogen activation on fibrin, the removal of carboxyl-terminal basic residues with carboxypeptidase B (CpB) results in the complete loss of the ability of cell surfaces to promote plasminogen activation [6].

Conformational changes induced by the interaction of plasminogen with cellular receptors also contribute to enhanced plasminogen activation on cell surfaces. It has been suggested that a major effect of the interaction of Glu-plasminogen with cell surfaces is to induce a conformational change in Glu-plasminogen that mimics the conformation of the more readily activated Lys-plasminogen, without the necessary *N*-terminal plasmic cleavage. However, recent studies indicate that the interaction of Glu-plasminogen with cell surfaces induces a conformational change that then promotes plasmic cleavage to the more readily activated Lys-plasminogen form [8,9,10,11].

On both the cell surface and on the fibrin surface, the formed plasmin is relatively protected from its major inhibitor, α_2_-antiplasmin [12,13] (Figure 1). As the initial interaction with plasmin is mediated via the *C*-terminal lysine of α_2_-antiplasmin [14], the protection from inhibition on cell surfaces is most likely due to the competition between the *C*-terminus of α_2_-antiplasmin and the *C*-terminal lysines exposed on cell surfaces.

The result of the foregoing interactions is the localization of the proteolytic activity of plasmin on cell surfaces, suggesting that when cells are located in the proximity of fibrin, cell surface plasminogen receptors have the potential to promote fibrinolysis in vivo.

## 3. Cellular Plasminogen Receptors

Numerous studies have addressed the molecular identity of plasminogen binding sites on eukaryotic cells. Consistent with the high capacity of cells for plasminogen and the diversity of cell types that interact with plasminogen, several cell surface molecules with plasminogen-binding function have been identified. CpB-sensitive cellular plasminogen receptors now comprise three categories: (1) a transmembrane protein synthesized with a *C*-terminal lysine and exposing a *C*-terminal lysine on the cell surfaces, plasminogen receptor KT (Plg-R_KT_) [15]; (2) proteins synthesized with *C*-terminal basic residues and lacking transmembrane domains, and having well-established intracellular functions, including α-enolase [16,17], cytokeratin 8 [18,19], S100A10 (in complex with annexin A2 within the annexin A2 heterotetramer (AIIt)) [20], histone H2B [21] and TIP49a [22], and (3) proteins that require proteolytic processing in order to expose a *C*-terminal basic residue to permit plasminogen binding, including actin [23,24,25]. A CpB-insensitive component of plasminogen binding is present on eukaryotic cells that does not promote plasminogen activation [6], which includes tissue factor [26] and non-protein gangliosides [27]. Additionally, transmembrane proteins (αIIbβ_3_ [28], α_M_β_2_ [29,30] and α_5_β_1_ [30], as well as amphoterin [31] and GP330 [32]) bind plasminogen, but are not synthesized with *C*-terminal basic residue, and whether these proteins undergo proteolysis to reveal *C*-terminal basic residues is not known. Specific review articles regarding the identification, functions and mechanisms of action of plasminogen receptors can be found in the volume introduced by reference [33].

The role of cell surface-associated plasmin in fibrinolysis in vivo is most likely carried out by plasminogen receptors exposing *C*-terminal lysines on the cell surfaces. Of this category, genetic deletion resulting in viable mice has been performed only for annexin A2 [34], S100A10 [35] and Plg-R_KT_ [36]. The assessment of the functions of other plasminogen receptors, H2B and α-enolase, has been carried out using antibody inhibition approaches [37,38].

## 4. Intravascular Fibrinolysis

Plasminogen-deficient mice exhibit severe spontaneous thrombosis in multiple organs [39,40]. The annexin A2/S100A10 heterotetrameric complex (AIIt) plays a clear role in regulating intravascular fibrinolysis, in both spontaneously arising thrombosis and challenge models [41]. The separate components of AIIt regulate each other’s expression [42,43,44,45]. Consequently, the genetic deletion of either annexin A2 or S100A10 in mice results in the deletion of the other component [44]. Annexin A2 deficiency results in spontaneous microvascular fibrin deposition in kidney, lung, heart, spleen liver and small intestine [34], while spontaneous fibrin deposition has been examined and demonstrated in the lung, liver and kidney of S100A10-deficient mice [35]. In addition, S100A10-deficient mice exhibit a four-fold reduction in bleeding time following tail clipping, consistent with decreased fibrinolysis of blood clots [35]. In contrast, Plg-R_KT_ deficiency does not have a significant effect on spontaneous fibrin deposition based on the examination of hematoxylin and eosin-stained tissues, including kidney, lung, liver, brain, heart muscle, skeletal muscle, lymheph nodes, thymus, spleen, stomach, small intestine, large intestine, colon, cecum, rectum, pancreas, adrenal, uterus, ovaries, vas deferens, testes, and epididymis [36], and genetic deletion of Plg-R_KT_ has no effect on bleeding time in response to tail clipping [46]. In contrast, Plg-R_KT_ deletion results in impaired fibrinolysis during wound healing and lactational development, as described below in Section 6 and Section 7. These differential effects on intravascular and tissue fibrinolysis may relate to differences in specific cell types present adjacent to and within fibrin clots in the local environment, as well as to the age of thrombi.

A role for AIIt in intravascular fibrinolysis is also demonstrated in challenge models. In response to FeCl_3_-induced carotid artery thrombosis, annexin A2-deficient mice exhibit impaired clearance of acute arterial thrombi [47]. In a batroxobin-induced clot lysis model, S100A10-deficient mice show a dramatic decrease in the ability to clear fibrin clots, compared with wild type controls [35]. These challenge models have not been tested in Plg-R_KT_-deficient mice to date.

## 5. Immune Cell Recruitment in the Inflammatory Response

Studies in plasminogen-deficient mice have revealed an essential role for plasminogen in macrophage and lymphocyte recruitment in the pro-inflammatory phase of the immune response [48,49,50,51]. Recent studies employing the thioglycollate-induced model of sterile peritonitis have established that impaired fibrinolysis is a major contributor to impaired macrophage recruitment in plasminogen-deficient mice [52]. Genetic deficiency of fibrinogen or the removal of the α_M_β_2_-binding domain of fibrinogen corrects the defect in macrophage recruitment in plasminogen-deficient mice [52]. These results were interpreted to suggest a requirement for fibrinolysis to disrupt the interaction of leukocytes with fibrino(ogen) via integrin α_M_β_2_ [52]. In addition, the removal of fibrin from the extravascular space also involves a plasmin-dependent endocytotic pathway on CCR2-positive macrophages [53].

The participation of plasminogen receptors that expose *C*-terminal basic amino acids on the cell surface is essential for optimal macrophage recruitment in sterile peritonitis [54]. Antibody inhibition approaches have revealed roles for H2B [37], α-enolase [37], and Plg-R_KT_ [55] in macrophage recruitment in this model, as well as a role for α-enolase in lung inflammation [38]. Genetic deletion has also revealed roles for S100A10 [56] and Plg-R_KT_ [36,57] in macrophage recruitment in sterile peritonitis. The direct effects of specific plasminogen receptors on fibrinolysis have not been investigated, but are likely to take place, based on the requirement for fibrinolysis in this model [52].

The participation of multiple plasminogen receptors in inflammatory macrophage recruitment is consistent with a muti-step process that may utilize distinct plasminogen receptors at different steps. Thus, traversing different barriers with distinct extracellular matrix compositions may require the involvement of distinct plasminogen receptors at distinct sites, and also requires fibrin(ogen)-independent steps. In addition to fibrinolysis and fibrin endocytosis, MMP9 activation is required for optimal plasminogen-dependent macrophage recruitment [58]. Notably, impaired activation of MMP9 is exhibited by S100A10-deficient macrophages in culture [56], as well as with the treatment of mice with an anti-Plg-R_KT_ monoclonal antibody in the sterile peritonitis model [55]. In addition, synthesis of the chemotactic cytokine, CCL2, that promotes macrophage recruitment, is impaired in models of immune cell recruitment in plasminogen-deficient mice [50,51], and Plg-R_KT_ deficiency results in decreased CCL2 synthesis [51]. Furthermore, the expression of distinct plasminogen receptors is likely to be distinct on distinct macrophage subtypes, as exemplified by the increased expression of Plg-R_KT_ on proinflammatory monocytes and macrophages [57].

## 6. Wound Healing

Wound healing is a complex and central pathophysiologic process that proceeds in overlapping, sequential steps [59]. In response to wounding, vascular permeability increases, leading to extravascular fibrin deposition in the wounded area as a key hemostatic event [60]. Subsequently, the proliferation phase of wound healing is characterized by fibrin clearance via fibrinolysis, and keratinocyte proliferation and migration through the fibrin-rich extracellular matrix [61].

Studies in plasminogen-deficient patients have documented an essential requirement for plasminogen in normal wound healing [62,63,64,65,66]. Impaired fibrinolysis in affected individuals leads to the formation of ligneous fibrin-rich pseudomembranes at mucosal sites during wound healing [67], most commonly seen in the conjunctivae and less frequently in the periodontal and gingival mucosae. In addition, the involvement of the mucosae of the respiratory tract and upper and lower gastrointestinal tracts can result in life threatening pseudomembranous obstruction [64].

Impaired wound healing in plasminogen-deficient mice was first demonstrated in an incisional skin wounding model [68]. Wounds from plasminogen-deficient mice were characterized by fibrin(ogen)-rich deposits in front of migrating keratinocytes, while fibrin(ogen) was uniformly present in wounds of wild type mice, leading to the conclusion that a major factor in the impaired wound healing in plasminogen-deficient mice is the decreased ability of keratinocytes to proteolytically dissect the fibrin-rich extracellular matrix [68]. Impairment in healing following myocardial infarction was also demonstrated in plasminogen-deficient mice, although fibrin deposition was not evaluated [69]. Impaired fibrinolysis is observed in several other models of wound healing in plasminogen-deficient mice. Plasminogen-deficient mice spontaneously develop otitis media with extensive fibrin deposition and abnormal keratin formation in the tympanic membrane, middle ear cavity and external ear canal [70]. Furthermore, several steps in the healing of perforated tympanic membranes, including the removal of fibrin, are impaired in these mice [71]. In concordance with the sites of impaired wound healing in plasminogen-deficient humans, plasminogen-deficient mice develop periodontitis with detachment of gingival tissue that is characterized by fibrin deposition [72]. Plasminogen is required for several steps of the wound healing process. Long after re-epithelialization, plasminogen-deficient mice exhibit extensive fibrin deposition in the wounded area, indicating inefficient debridement, thus emphasizing an additional role for plasminogen in the termination of the wound healing response [73]. In a model of radiation injury, fibrin deposition is present in the wound, and treatment with plasminogen accelerates healing and removes fibrin deposits [74]. In view of the many examples of impaired fibrin clearance in plasminogen-deficient mice, it is most noteworthy that the healing defect in plasminogen-deficient mice is corrected by the genetic deletion of fibrinogen [75], leading to the suggestion that the primary function of plasminogen in wound healing is fibrinolysis [76].

**Figure 2:**
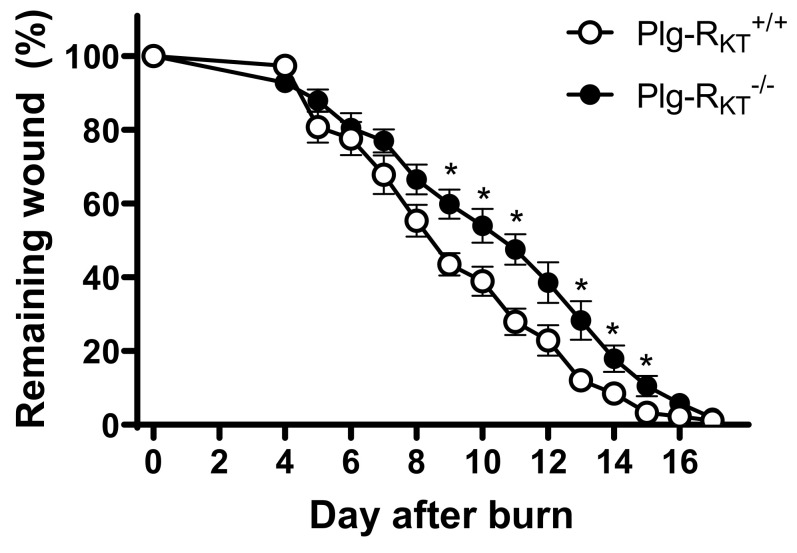
Effect of Plg-R_KT_ deficiency on burn wound healing. (A) Quantification of the remaining wound area at different time points after wounding of male Plg-R_KT_^−/−^ mice (●) and Plg-R_KT_^+/+^ litter mates (○) (*n* = 6). The mixed effects model (REML) showed a significant effect for time (*p* < 0.0001, f = 396.6), and for genotype (*p* = 0.040, f = 6.00), and a significant time X genotype interaction (*p* < 0.0001, f = 4.289). Post hoc testing was done with 2-tailed *t* tests at each time point * *p* < 0.05. This figure is modified from a figure originally published in [80]. Creative common license available at: http://creativecommons.org/licenses/by/4.0/.

**Figure 3:**
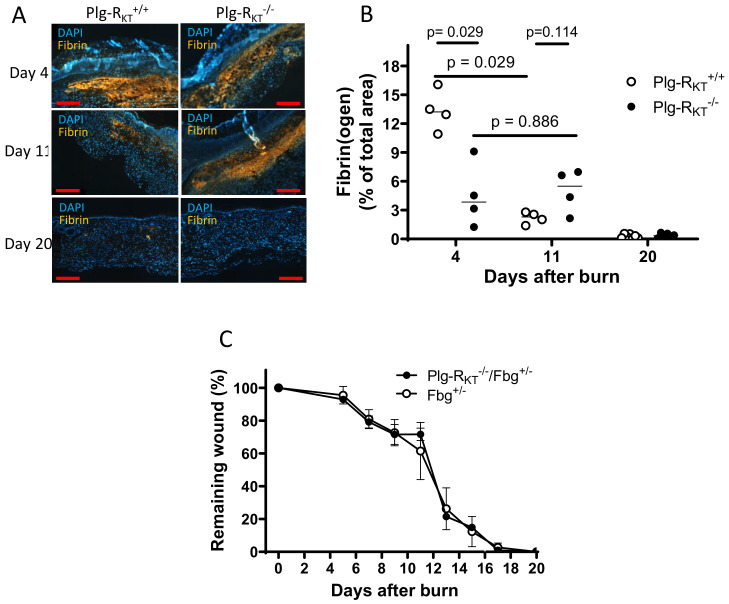
Enhanced fibrin(ogen) content in Plg-R_KT_^−/−^ wound tissue and effect of fibrinogen heterozygosity on burn wound healing. (**A**) Immunohistochemical staining for fibrin in the tissue of Plg-R_KT_^−/−^ and Plg-R_KT_^+/+^ mice collected at different time points after induction of burn wounds (*n* = 4). Scale bar = 200 µm. (**B**) Quantification of fibrinogen area in Panel A (%) based on immunostaining using Image J software https://imagej.nih.gov/ij/download.html. (**C**) Quantification of the remaining wound area at different time points after burn wounding of male Plg-R_KT_^−/−^/Fgn^+/−^ (*n* = 4) mice and Plg-R_KT_^+/+^/Fgn^+/−^ mice (*n* = 5). Post hoc testing was done by Mann–Whitney test. This figure is modified from a figure originally published in [80]. Creative common license available at: http://creativecommons.org/licenses/by/4.0/.

Recent studies have demonstrated a novel key role for plasminogen in wound healing, in addition to orchestrating fibrinolysis. Plasminogen binds to macrophages and neutrophils and is transported to the wound area, where the level of plasminogen is locally increased. This induces intracellular signaling and cytokine release [76]. Notably, the recruitment of immune cells to cutaneous wounds is not affected by plasminogen deficiency [68,73,76] and thus, the role of plasminogen in the initial stages of inflammation is predominantly the induction of intracellular signaling. Earlier studies in vitro documented the plasmin-dependent stimulation of intracellular signaling pathways, and cytokine release by monocytes and macrophages that depends on the interaction of plasmin with cell surfaces [77,78,79]. Notably, the deletion of Plg-R_KT_ in mice (Plg-R_KT_^−/−^ mice) leads to a significant delay in cutaneous wound healing (Figure 2) [80]. Consistent with studies in plasminogen-deficient mice, the rate of fibrin clearance is markedly impaired in wounds of Plg-R_KT_^−/−^ mice (Figure 3A,B) [80]. Moreover, the genetic reduction of fibrinogen levels by 50% in Plg-R_KT_^−/−^ mice corrects the wound healing impairment in these mice (Figure 3C) [80].

Plg-R_KT_ deletion also impairs immune cell function in wound healing, although there is no significant effect on immune cell recruitment to the wound site [80] (similar to results with plasminogen-deficient mice [68,73,76]). The injection of mice with an anti-Plg-R_KT_ blocking monoclonal antibody impairs plasminogen transport to the wound area [80]. Furthermore, the specific deletion of Plg-R_KT_ in myeloid cells results in a prominent and highly significant delay in wound healing [80].

**Figure 4:**
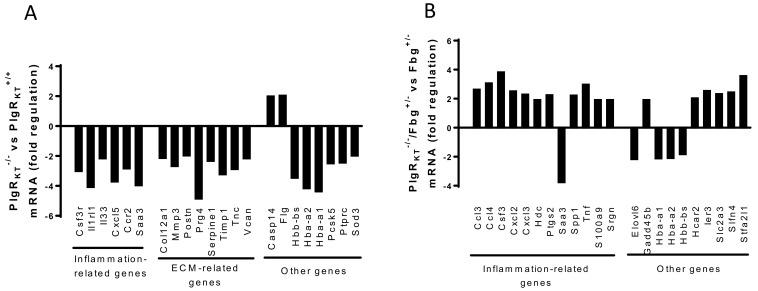
Effect of Plg-R_KT_ deletion on gene expression in wound tissue. Gene expression was studied using mRNA sequencing in samples taken at day 11 (*n* = 4). All the genes had corrected *p*-value ≤ 0.05. (**A**) Genes whose expression changed ≥ 1.5-fold in Plg-R_KT_^−/−^ compared with Plg-R_KT_^+**/**+^ tissue. (**B**) Genes whose expression changed ≥ 1.5-fold in Plg-R_KT_^−/−^/Fibrinogen^+/−^ compared with Plg-R_KT_^+/+^/Fibrinogen^+/−^ tissue. This figure is modified from a figure originally published in [80]. Creative common license available at: http://creativecommons.org/licenses/by/4.0/.

A comparison of gene expression profiles in wound tissue of Plg-R_KT_^−/−^ mice and their wild type littermates showed the downregulation of six genes participating in the inflammatory response, including cytokines, and eight genes related to the extracellular matrix (Figure 4A) [80]. Remarkably, in Plg-R_KT_^−/−^ mice with genetic heterozygosity for fibrinogen, the expression of eleven inflammation-related genes was upregulated, while these genes were not upregulated in Plg-R_KT_^−/−^ wound tissue on a fibrinogen wild type background (Figure 4B) [80].

Thus, Plg-R_KT_ regulates wound healing via promoting fibrinolysis as well as regulating plasminogen-dependent cytokine release and intracellular signaling. Moreover, these latter functions are related by the effects of fibrin(ogen) on intracellular signaling mediated by Plg-R_KT_.

## 7. Lactation

**Figure 5:**
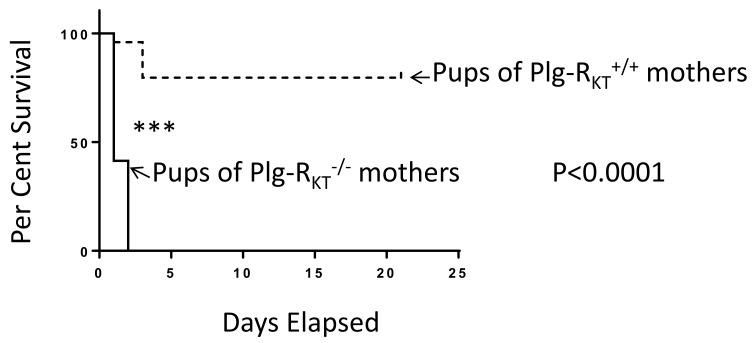
Plg-R_KT_ null mice cannot successfully lactate. Survival data are shown for a cohort of offspring of Plg-R_KT_^+/+^ (dashed lines) and Plg-R_KT_^−/−^ (solid lines) primiparous female littermates: 48 offspring of Plg-R_KT_^+/+^ mice and 30 offspring of Plg-R_KT_^−/−^ mice. **** *p* < 0.0001 Log-rank (Mantel Cox test). This research was originally published in Miles, L.A.; Baik, N.; Bai, H.; Makarenkova, H.P.; Kiosses, W.B.; Krajewski, S.; Castellino, F.J.; Valenzuela, A.; Varki, N.M.; Mueller, B.M.; Parmer, R.J. The plasminogen receptor, Plg-RKT, is essential for mammary lobuloalveolar development and lactation. *J. Thromb. Haemost.* **2018**, *16*, 919–932 [83], and is reprinted with permission from John Wiley and Sons.

**Figure 6:**
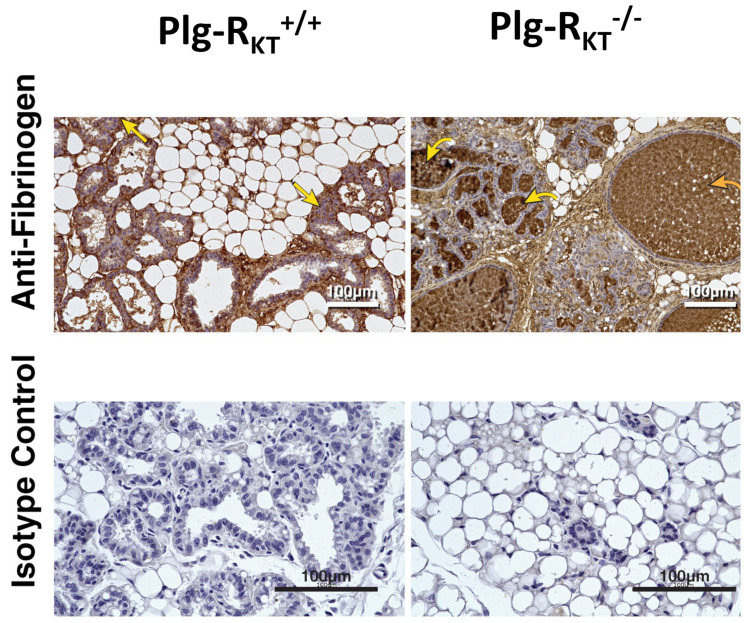
Plg-R_KT_ deletion alters the ECM in lactating mammary glands. Abdominal mammary glands were harvested from Plg-R_KT_^+/+^ and Plg-R_KT_^−/−^ female mice two days postpartum and stained with anti-fibrin(ogen) antibody (the curved yellow arrow labels represent fibrin deposition in alveoli; the curved orange arrow labels represent fibrin deposition in dilated ducts; the straight yellow arrows labels represent fibrin deposition in adipose tissue) or non-immune rabbit IgG control. This research was originally published in Miles, L.A.; Baik, N.; Bai, H.; Makarenkova, H.P.; Kiosses, W.B.; Krajewski, S.; Castellino, F.J.; Valenzuela, A.; Varki, N.M.; Mueller, B.M.; Parmer, R.J. The plasminogen receptor, Plg-RKT, is essential for mammary lobuloalveolar development and lactation. *J. Thromb. Haemost.* **2018**, *16*, 919–932. [83] Reprinted with permission from John Wiley and Sons.

Plasminogen plays an essential role in lactational development in the mouse mammary gland [81,82]. Whereas 60% of offspring of plasminogen-deficient mice are alive at postpartum day 2, 75% of pups die by postpartum day 17 [82]. Although milk synthesis is not affected, fibrin deposition is noted in the ducts of plasminogen-deficient mice, consistent with the blockade of ducts, preventing the release of milk. Consequently, the lactation defect is rescued when plasminogen-deficient mice are bred into a heterozygous fibrinogen background [82]. Plg-R_KT_^−/−^ mice exhibit a more severe lactation defect in which all offspring of Plg-R_KT_^−/−^ mice die by postpartum day 2 (Figure 5) [83]. A massive accumulation of fibrin is present in the alveoli and ducts of these mice (Figure 6) [83].

**Figure 7:**
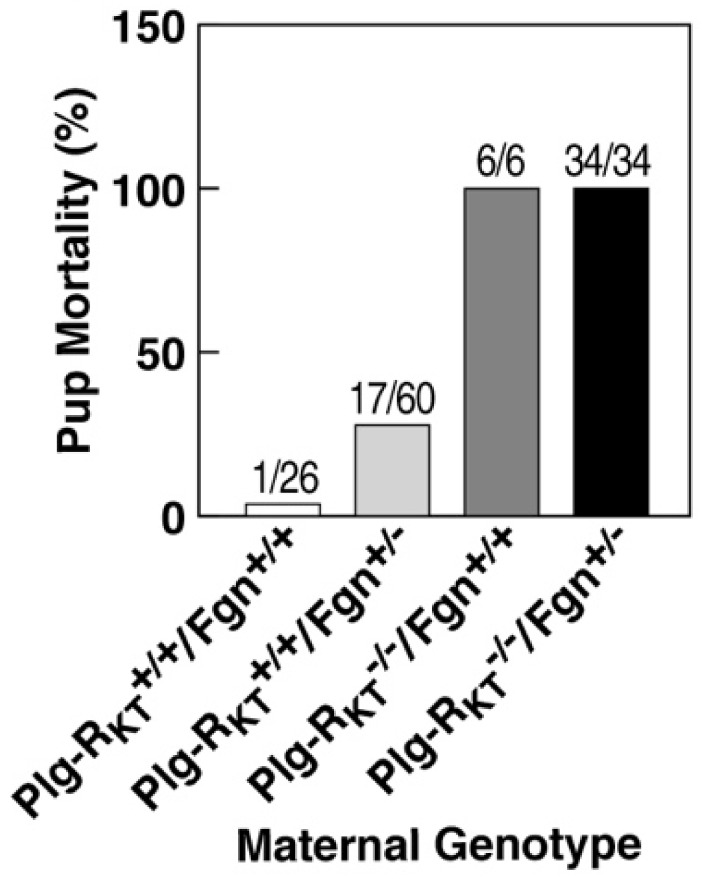
Effect of fibrinogen heterozygosity on alveolar development and lactational competence in Plg-R_KT_^−/−^ mice. Pup mortality at day 2 postpartum as a function of maternal genotype. This research was originally published in Miles, L.A.; Baik, N.; Bai, H.; Makarenkova, H.P.; Kiosses, W.B.; Krajewski, S.; Castellino, F.J.; Valenzuela, A.; Varki, N.M.; Mueller, B.M.; Parmer, R.J. The plasminogen receptor, Plg-RKT, is essential for mammary lobuloalveolar development and lactation. *J. Thromb. Haemost.* **2018**, *16*, 919–932. [83] Reprinted with permission from John Wiley and Sons.

However, pup survival is not rescued by fibrinogen heterozygosity (Figure 7) [83]. Thus, in addition to an effect on fibrinolysis, the deletion of Plg-R_KT_ is likely to exert other plasminogen- and fibrin(ogen)-independent effects on lactational development. These include the downregulation of EGF synthesis and, consequently, the downregulation of the pro-survival protein, Mcl-1, leading to increased apoptosis and decreased alveolar development [83]. The phenotypes of plasminogen and Plg-R_KT_^−/−^ mammary glands are also distinct, in that plasminogen deficiency results in delays in ductal development during puberty [82] that are not observed in Plg-R_KT_^−/−^ glands [83]. Recently, the loss of S100A10 has been shown to impair ductal development during the early stages of mammary development [84]. Thus, mammary development provides an example of the utilization of multiple plasminogen receptors at distinct steps in a physiologic process.

## 8. Conclusions

Emerging evidence demonstrates a role for specific plasminogen receptors in fibrinolysis in both physiological and pathological conditions in vivo. In addition, fibrin(ogen) appears to reciprocally modulate the function of plasminogen receptors. Results have been obtained by the genetic deletion of specific receptors and through the use of function-blocking antibodies. Results with antibody blockade are more limited, most likely due to the expense and the requirement for repeated injections in these in vivo experiments. Plasminogen receptors exposing *C*-terminal lysines on cell surfaces that have been genetically deleted to date are components of the AIIt complex (annexin A2 and S100A10) and Plg-R_KT_. The deletion of plasminogen receptors that are synthesized with *C*-terminal basic residues, lack transmembrane domains and have additional essential intracellular functions is likely to result in non-viable mice. The creation of transgenic mice bearing a plasminogen receptor with a *C*-terminal K to A substitution (that is not predicted to interact with plasminogen) should yield informative results in the future. In addition, the investigation of the functions of AIIt and Plg-R_KT_ in other fibrinolysis-dependent functions that are impaired in plasminogen-deficient mice should yield further information regarding the in vivo roles of these plasminogen receptors in fibrin surveillance.

## Data Availability

Not applicable.
